# Impact of rice wine-steamed *Cistanche deserticola* polysaccharides on intestinal flora and immunological modulation in immunosuppressive mice induced by cyclophosphamide

**DOI:** 10.3389/fimmu.2026.1732818

**Published:** 2026-02-05

**Authors:** Jing Lian, Yuexin Zhu, Qiushi Hu, Kexu Dong, Zihang Peng, Yurou Feng, Na Ling, Caina Yu, Pengpeng Liu, Xuefei Lei, Ji Shi, Tianzhu Jia

**Affiliations:** 1 Liaoning University of Traditional Chinese Medicine, Department of Pharmacy, Dalian, Liaoning, China; 2 Anhui Jiren Pharmaceutical Co., Ltd, Anhui, China; 3 Key Research Laboratory of Traditional Chinese Medicine Processing Process Principles of the State Administration of Traditional Chinese Medicine, Dalian, Liaoning, China; 4 Liaoning Provincial Traditional Chinese Medicine Processing Professional Technology Innovation Center, Dalian, Liaoning, China

**Keywords:** CD4+/CD8+, *Cistanche deserticola* polysaccharide, cyclophosphamide, gut microbiota, immune regulation, short-chain fatty acids

## Abstract

**Introduction:**

*Cistanche deserticola*, a valued medicinal and food homologous material in traditional Chinese medicine, is renowned for enhancing immunity and protecting the mucosal barrier, with polysaccharides considered its primary active components. Raw material requires processing for optimal efficacy, yet most studies focus on unprocessed polysaccharides (RCP). This study investigates how traditional rice-wine processing alters the polysaccharides (WCP) and their immunomodulatory effects.

**Methods:**

The physicochemical properties of RCP and WCP were characterized using SEM, HPGPC, FT-IR, and GC-MS. Immunosuppressed mice induced by cyclophosphamide (CTX) were used as an *in vivo* model. Immune function was assessed via body weight, organ indices, serum immunoglobulins (IgA, IgM), cytokines (IL-2, IFN-γ), spleen histopathology, and T cell subsets (CD4+/CD8+). Gut microbiota composition was analyzed by 16S rRNA sequencing, and short-chain fatty acid (SCFA) levels were measured.

**Results:**

Wine processing significantly modified the polysaccharides, increasing polysaccharide content, markedly reducing molecular weight, and altering monosaccharide composition in WCP. In CTX-induced mice, WCP administration showed superior immunomodulatory effects: it significantly improved spleen and thymus indices, elevated serum IL-2, IFN-γ, IgA, and IgM levels, and increased the CD4+/CD8+ ratio. Furthermore, WCP restored CTX-disturbed intestinal SCFA levels and positively modulated gut microbiota by increasing Bacteroidetes and beneficial probiotics, lowering the *Firmicutes/Bacteroidetes* ratio, and suppressing pathogenic bacteria.

**Discussion:**

Traditional rice-wine processing optimizes the molecular structure of Cistanche polysaccharides and significantly enhances their immunomodulatory efficacy in a cyclophosphamide-induced immunosuppression model. The enhanced activity is associated with marked improvements in gut microbiota composition and systemic immune parameters, suggesting involvement of the gut-immune axis. These findings provide a scientific basis for the traditional processing method and support the further development of processed Cistanche as a potential functional food or therapeutic agent.

## Graphical Abstract

**Figure:**
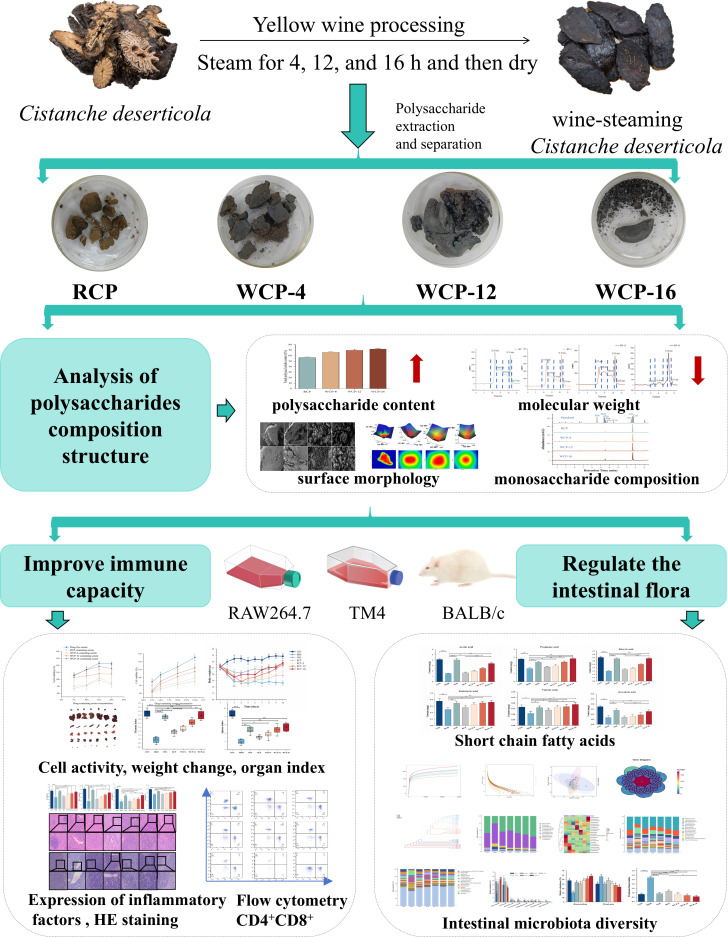

## 1 Introduction

The intestinal microbiota plays a pivotal role in regulating host immunity, with dysbiosis being implicated in a wide range of immune-mediated disorders. Dietary fibers and plant-derived polysaccharides, which resist host digestion, serve as critical fermentable substrates for commensal bacteria, thereby shaping microbial ecology and function (1). Through the production of metabolites such as short-chain fatty acids (SCFAs), these microbial communities directly and indirectly modulate intestinal barrier integrity, local immune responses, and systemic inflammation (2, 3).

This gut-immune axis represents a promising therapeutic target for immune dysfunction. Cyclophosphamide (CTX), a potent alkylating agent, is a well-established inducer of immunosuppression in preclinical models, reliably recapitulating leukopenia, immune organ atrophy, and functional impairment (4). Its mechanism extends beyond direct lymphocytotoxicity to encompass profound gastrointestinal damage. CTX disrupts the intestinal epithelial barrier, depletes beneficial SCFA-producing bacteria, and induces a state of microbial dysbiosis (5). This breach of intestinal homeostasis facilitates bacterial translocation, which in turn exacerbates systemic inflammation and creates a vicious cycle that further aggravates immune suppression (6).

Polysaccharides derived from various plants have shown promise in modulating this axis. *Cistanche deserticola* Y.C.Ma, a desert plant, is a rich source of such polysaccharides, which are reported to exhibit immunomodulatory and potential prebiotic activities (7, 8). The bioactivity of polysaccharides is intrinsically linked to their physicochemical structure, which can be deliberately modified through processing techniques like heat treatment (9). Preliminary evidence suggests that steam-processing alters the structural properties of *Cistanche deserticola* polysaccharides (10), but a systematic investigation into how processing duration affects their structure and, consequently, their function in immune modulation remains unaddressed. Nonetheless, the majority of recent research has mostly concentrated on the pharmacological effects and isolation of Cistanche’s chemical constituents (11). Comparatively few systematic investigations have been conducted on the patterns and changes in Cistanche’s polysaccharide content following varying steaming times.

A critical gap exists in current knowledge. While the immunomodulatory effects of plant polysaccharides are recognized, a clear mechanistic link is missing between processing-induced structural modifications, their consequent impact on rescuing CTX-induced gut dysbiosis, and the final immune restorative outcomes. We hypothesize that steam-processing optimizes the structure of Cistanche deserticola polysaccharides, thereby enhancing their efficacy in ameliorating CTX-induced immunosuppression via restoration of the gut microbiota and the SCFAs pathway. To test this, polysaccharides were extracted from *Cistanche deserticola* subjected to varying steam-processing durations. Their immunorestorative capacity was evaluated in a CTX-induced murine model, with a focused analysis on gut microbiota recomposition, SCFA production, and systemic immune parameters. This study aims to establish a processing-dependent structure-activity relationship and to elucidate the associated gut-mediated immunomodulatory mechanisms.

## 2 Materials and methods

### 2.1 Materials

The cultivated crude drug of *Cistanche deserticola* Y.C. Ma was sourced from the Alxa League, Inner Mongolia, China (Coordinates: 105.65, 38.87). The material from the 2024 crop year (Batch No. 20240515) was authenticated by Prof. Yanjun Zhai at Liaoning University of Traditional Chinese Medicine based on its morphological characteristics (dry fleshy stem with slimy leaflets). A voucher specimen derived from this batch has been deposited at the Herbarium of Liaoning University of Traditional Chinese Medicine under the accession number [LUTCM-P-2024-015]. The authenticated material was dried and stored in airtight containers prior to extraction. For the reference materials, glucans with molecular weights of 180, 2700, 5250, 9750, 13050, 36800, 64650, 135350, and 2000000 Dalton were procured from the Reference Materials Research Center of the National Institute of Metrology. Additionally, the following monosaccharides, all with a purity exceeding 98%, were purchased from Shanghai Yuan Ye Biotechnology Co., Ltd.: glucose (Glu, batch number: PS 020418), galactose (Gal, batch number: wkq 18101505), mannose (Man, batch number: PS 3519-0020), arabinose (Ara, batch number: wkq 19030109), rhamnose (Rha, batch number: PS 1383-0100), and fructose (Fru, batch number: wkq 16062201).Cyclophosphamide (CTX, NO.C8685) and Levamisole Hydrochloride (LH, NO.L8230) were obtained from Beijing Solibo Technology Co., Ltd. ELISA kits for IL-2 (NO.MM-0701M1), IFN-γ (NO.MM-0182M1), IgM (NO.MM-0058M1), and IgA (NO.MM-0055M1) were sourced from Jiangsu Enzyme Immunochemical Industry Co., Ltd. FITC Anti-Mouse CD4 Antibody (NO. AF21880), APC Anti-Mouse CD8 Antibody (NO. AF19993), ACK Lysis Buffer (E-CK-A105), and Cell Staining Buffer (E-CK-A107) were purchased from Elabscience Wuhan Elireit Biotechnology Co., Ltd. Trifluoroacetic acid (TFA) and other reagents used in this study were of analytical grade.

### 2.2 Preparation of polysaccharides

#### 2.2.1 Preparation of Cistanche samples

##### 2.2.1.1 Raw Cistanche decoction pieces

Raw Cistanche was steamed for 20 minutes, cut into 3 mm-thick slices, and air-dried to constant weight.

##### 2.2.1.2 Wine-processed Cistanche decoction pieces

Raw Cistanche pieces were first soaked in 30% rice wine (10:3 w/v) for 8 hours to allow sufficient absorption of the wine. Then steamed for 4 h, 12h, and 16 h, respectively. After steaming, the processed pieces were dried in a forced-air oven at 50°C until completely desiccated (12).

#### 2.2.2 Polysaccharides extraction and purification

##### 2.2.2.1 Pretreatment and defatting

Slices of dry RCD, WCD-4, WCD-12, and WCD-16 were macerated in 95% alcohol (1:10 w/v) for two weeks, shaking occasionally, to defatten them. To get a homogenous powder, the defatted slices were crushed, passed using a 65-mesh sieve, air-dried to eliminate any remaining ethanol.

##### 2.2.2.2 Hot water extraction

Defatted powder was combined with 1:10 w/v deionized water and refluxed for three hours at 100°C. After centrifuging the extract for 15 minutes at 4,000 rpm to get rid of any insoluble residues, the leftover liquid was gathered.

##### 2.2.2.3 Dialysis with ethanol precipitation

To get rid of low-molecular-weight contaminants, the basic extract (MWCO = 3,500 Da) underwent dialysis for 48 hours against ionized water. After being concentrated at lower pressure, the retentate was combined with anhydrous ethanol to a volume percentage of 80% (v/v). Overnight chilling at 4°C precipitated polysaccharides.

##### 2.2.2.4 Isolation and deproteinization

Precipitated polysaccharides were collected by centrifugation (8,000 rpm, 20 min, 4°C), washed thrice with anhydrous ethanol and acetone, and freeze-dried to obtain crude fractions (crude RCP, WCP-4, WCP-12, WCP-16). Deproteinization was performed using the Sevag method: a 2% (w/v) crude polysaccharide solution was mixed with Sevag reagent (chloroform:n-butanol = 4:1 v/v) at a 5:1 (solution:reagent) ratio, vortexed for 30 min, and centrifuged (4,000 rpm, 15 min). The deproteinization cycle was repeated until no proteinaceous interphase was visible for two consecutive cycles. To provide a quantitative endpoint, protein removal was confirmed by monitoring the absorbance of the aqueous layer at 280 nm (A_280_) after each cycle, and the process was terminated when A_280_< 0.10. The deproteinized supernatant was then collected, redialyzed against distilled water, freeze-dried, and stored at -20 °C for subsequent analysis.

### 2.3 Polysaccharide content determination

Determination of Total Polysaccharide Content (Phenol-Sulfuric Acid Method): The total polysaccharide content was quantified using the phenol-sulfuric acid method with UV-Vis spectrophotometric detection. Briefly, a calibration curve was established using anhydrous D-glucose as the standard. A series of standard solutions were prepared at concentrations of 0, 20, 40, 60, 80, and 100 µg/mL. Each standard (or sample) was reacted with 5% (v/v) aqueous phenol and concentrated sulfuric acid. After cooling, the absorbance was measured at 490 nm. The standard curve, plotting absorbance against glucose concentration, demonstrated excellent linearity within the range of 0–100 µg/mL. The polysaccharide content in test samples was calculated by interpolating their absorbance values onto this calibration curve.

### 2.4 Determination of morphological structure

Polysaccharide samples were sieved through a 100-mesh screen. A small quantity was placed on conductive carbon tape, sputter-coated with gold, and then scanned using an electron microscope. The operational parameters were as follows: SEM mode; acceleration voltage range, 0.02~30 kV; beam current range, 12 pA~100 nA; thermal field emission Schottky electron gun with beam stability better than 0.2%/h; an in-lens secondary electron detector (in-lens Duo) and an Everhart-Thornley secondary electron detector were employed. The sample chamber dimensions were 330 mm ×270 mm, and the magnification factors used were ×500 and ×5000.

Micro-infrared instrument with OMNIC v9.0 infrared spectrum analysis software (ThermoFisher Nicolet iS50/iN10) was poured into liquid nitrogen, and the fine powder of the polysaccharide sample was dried through a 150-mesh sieve and gently blown on a gold mirror. The experimental conditions were selected (in the reflection mode, the spectral acquisition was 4 000~400 cm^-1^, the cumulative scanning was 64 times, the scanning was 32 s, and the resolution was 4 cm^-1^). Infrared imaging was used to perform point-to-point mapping on the selected sample area, and then the sample was scanned.

### 2.5 Molecular weight determination

With modifications based on the method of Li et al., the molecular masses of the polysaccharides were determined by High-Performance Gel Permeation Chromatography (HPGPC) (13). The analysis was performed on an Agilent 1260-LC system equipped with a refractive index detector (RID), using a Nippon Shokubai TSKgel G4000PWXL column (300 mm × 7.8 mm, 10 µm). RCP and WCP samples (4 mg) were dissolved in 1.0 mL of ultra-pure water and filtered through a 0.22 µm membrane. Dextran standards were used for calibration. The chromatographic conditions were as follows: column temperature, 45°C; injection volume, 5 µL; flow rate, 0.5 mL/min; mobile phase, 150 mM phosphate buffer (pH 7.0). A calibration curve was established by plotting the logarithm of the molecular weight (log Mw) of the dextran standards against their retention time (t).

### 2.6 Monosaccharide components analysis

The composition of monosaccharides was determined by gas chromatography. The analysis was carried out using the internal standard method for quantification, with inositol as the internal standard reference substance. The acquisition and analysis of chromatographic data were completed through the Agilent-Mass Hunter workstation. Precisely weigh 2.00 mg of the polysaccharide samples obtained from different steaming times into an ampoule. Dissolve them in 2 M trifluoroacetic acid solution, seal the bottle, and hydrolyze at 120°C for 3 h. Remove the sample, let it cool. Centrifuge at 3500 r/min for 15 min, aspirate the supernatant, perform vacuum concentration, dissolve in methanol, dry, repeat the process until all the residual trifluoroacetic acid is removed. Then, dry to obtain the product. Dissolve the above hydrolyzed Rhodiola rosea polysaccharides with 3.00 mL of ultrapure water. Add 30 mg of borohydride, and shake at room temperature overnight. Gradually add a small amount of acetic acid multiple times until the pH is 6. vacuum concentration. Add 6.00 mL methanol to mix and concentrate, repeat 6 times, until there is no white solid on the tube wall, and dry in the oven at 110°C. The above reduction products were added to 1.50 mL acetic anhydride and 1.50 mL pyridine, and the reaction was carried out in a sealed bottle at 110°C for 3 h, oscillating constantly until there was no precipitation. After vacuum concentration, 5.00 mL chloroform was added to dissolve, washed with 5 mL ultrapure water, repeated for 6 times, and the aqueous phase was discarded. Anhydrous sodium sulfate was added to the chloroform layer to clarify. After filtration with 0.22 μm organic membrane, it was sealed and stored.

Containing glucose (Glu), galactose (Gal), mannose (Man), arabinose (Ara), rhamnose (Rha) and fructose (Fru), the concentrations were 10.00 mg/mL, 5.00 mg/mL, 2.5 mg/mL, 1.00 mg/mL, 0.25 mg/mL and 0.10 mg/mL, respectively. 250 μg inositol (internal standard) and 5.00 mg monosaccharide standard were accurately measured, respectively. After sodium borohydride reduction and derivatization, they were filtered by 0.22 μm organic membrane and sealed for preservation. The Agilent equipment with an HP-5 MS-UI capillaries column (30 m × 0.250 mm × 0.25 μm) and a nitrogen carrier gas with a flow speed of 1.00 mL/min; carrier gas: nitrogen, flow rate of 1.00 mL/min; injection volume: 1 μL; not shunted. Temperature programming (initial temperature of 120°C, 5°C/min to 200°C; the temperature was increased from 2°C/min to 202°C/min and maintained for 1 min. 2°C/min to 215°C/min; to 20°C/min to 270°C); the temperature of injector and detector was 250°C,Ion source with a temperature of 230 °C, quadruple warmth of 150°C, electron bombarding ion source (EI), and electron collision energy of 70 eV, acceleration voltage 34.6 V, multiplier voltage 1.388 kV, resolution 2500, scanning range *m/z*: 40–450 amu, scanning time: 331 ms.

### 2.7 Preparation of drug-containing serum

Thirty male SD rats weighing between 200 and 220 grams, SPF grade, from Liaoning the Changsheng Biotech Co., Ltd. were split into five groups at random: control (The control group was given 0.9% normal saline,200 mg/kg), RCP(200 mg/kg), WCP-4(200 mg/kg), WCP-12(200 mg/kg), and WCP-16(200 mg/kg). For a whole week, they received the medications nonstop. Blood was drawn from the abdominal aorta and anesthesia was administered one hour following the last intragastric injection. The animals were anesthetized with 4% isoflurane [inhaled].After 15 minutes of centrifuging the blood at 2000 rpm, the serum was isolated and deactivated for 30 minutes at 56°C. After passing through a 0.45-micron filter, it was divided and kept at -80°C for storage. After each experiment, all the animals were euthanized by inhaling high-concentration CO_2_ (at a flow rate of 30% volume displacement per minute).The Liaoning University of Traditional Chinese Medicine’s Animal Ethics Committee examined and authorized each and every animal experiment.(Approval Number: 2025067).

### 2.8 Cell culture

We bought mouse Sertoli TM4 cells (cell line no. STCC20050P) and mouse monoclonal macrophages RAW264.7 cells (cell line no. STCC20020P) from Wuhan Zixian Biotech Co., Ltd. (Wuhan, China).

#### 2.8.1 Culture conditions for TM_4_ cells

TM4 cells were cultured in DMEM/F12 medium supplemented with 2.5% fetal bovine serum, 5% horse serum and 1% penicillin-streptomycin. Cells were maintained at 37°C with 5% CO_2_ in a humidified incubator, and passaged at 1:2 split ratio upon reaching 60-70% confluence.

#### 2.8.2 Culture conditions for RAW264.7 cells

RAW264.7 cells were cultivated in high-glucose in Dulbecco’s Modified Eagles Medium (DMEM), which was enhanced with 1% penicillin-streptomycin (Sorlab, China) and 10% fetal bovine serum (FBS; Gibco). Equal to TM4 cells, the cells were kept in a humidified incubator at 37°C with 5% CO_2_. When they reached 85% confluence, they were subcultured at a 1:3 split ratio.

### 2.9 Cell grouping and CCK-8 assay for cell viability

Logarithmic-phase RAW264.7 and TM4 cells were grown in a CO_2_ incubator after being separately put into 96-well plates at an average density of 2×10^5^ cells/mL (100 μL per well). Rat serum at different concentrations was added to serum-starved cells (FBS-free medium) the next day. These cells were then split into seven groups: blank serum group, RCP serum group, WCP-4 serum group, WCP-12 serum group, WCP-16 serum group, positive control group (cells added medium with no rat serum), and negative control group (medium just). Following a 12-hour serum intervention, 100 μL of CCK-8 solution (Sorlub, E-CK-A362) was administered to each well after the medium had been aspirated. After that, the plate was incubated for two more hours in the dark. Lastly, a microscope was used to measure the optical density (OD) at 450 nm.


$$Cell\;viability\;(\%)=\frac{OD_{medication\;ad\min istration\;treatment\;group}-OD_{negative\;control\;group}}{OD_{positive\;control\;group}-OD_{negative\;control\;group}}$$

### 2.10 Animals and drug administration

**Figure 1:**
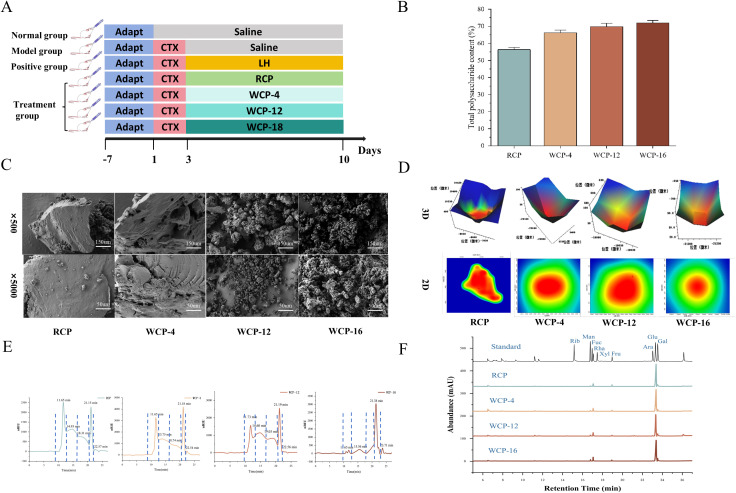
**(A)** Flow chart of animal model. **(B)** Determination of polysaccharide content in *Cistanche deserticola* with the extension of steaming time. The data are expressed as the mean standard deviation (n=3). **(C)** Observation of SEM surface morphology of polysaccharide. **(D)** Observation of microscopic infrared surface morphology of polysaccharide. **(E)** Determination of molecular weight. **(F)** monosaccharide composition.


Following a one-week acclimation period, seventy 6-week-old male Balb/c mice were randomly assigned to seven groups (n=10/group): control, model, positive control (levamisole hydrochloride), RCP, WCP-4, WCP-12, and WCP-16. To establish an immunosuppressive model (14), mice in all groups except the control received intraperitoneal injections of cyclophosphamide (80 mg/kg body weight) for three consecutive days (**Figure 1A**).Post-modeling, treatment groups (RCP, WCP-4, WCP-12, WCP-16) received daily oral gavage of their respective test substances (200 mg/kg/day). The positive control group received levamisole hydrochloride (80 mg/kg/day) via gavage. Both the control and model groups received an equivalent volume of normal saline daily. Treatment continued for 7 days, during which body weight was monitored. On the final day of the experiment (after the 7-day treatment period and prior to euthanasia), fresh fecal pellets were collected individually from each mouse in all groups immediately upon spontaneous defecation or by gentle abdominal massage. These individual fecal samples were immediately frozen and stored at -80°C for subsequent gut microbiota analysis. Blood samples were collected from the inner can thus, mice were then euthanized, and spleen and thymus organs were excised. These organs were rinsed with ice-cold normal saline, gently blotted dry on filter paper, and weighed to calculate organ indices. Calculate organ index (15).

$$\text{Spleen index}\;=\frac{\text{Spleen weight }\left(\text{mg}\right)}{\text{Body weight }\left(\text{g}\right)}\;$$

$$\text{Thymus index }=\frac{\text{Thymus weight }\left(\text{mg}\right)}{\text{Body weight }\left(\text{g}\right)}$$

#### 2.10.1 Assessment of humoral immunity by ELISA

To assess the impact of RCP and WCP on the humoral immune system, serum levels of immunoglobulins (IgA, IgM) and inflammatory cytokines (IL-2, IFN-γ) were measured. Serum samples from all animals in each group (n = 10) were analyzed. For each target analyte, 50 μL of serum was used according to the manufacturer’s instructions of the respective commercial ELISA kits. Absorbance was measured using a microplate reader, and concentrations were calculated based on a standard curve.

#### 2.10.2 Histopathology

Portions of spleen and thymus tissue were fixed in 4% paraformaldehyde, dehydrated through a graded ethanol series, paraffin-embedded, and sectioned at a thickness of 4 µm for subsequent hematoxylin and eosin (H&E) staining. This analysis was performed on tissue samples collected from all animals at the experimental endpoint, with a sample size of n = 10 per group. Morphological assessment was carried out by experienced pathologists who were blinded to the group assignments.

#### 2.10.3 Flow cytometry (T lymphocyte subsets)

Analysis of T lymphocyte subsets was performed on whole blood samples from a subset of mice (n = 6 per group), randomly selected from each cohort. Briefly, Heparinized whole blood was diluted with PBS. Erythrocytes were lysed by incubation with prechilled red blood cell lysis buffer (4°C). After centrifugation (1500 rpm, 5 min), the lymphocyte pellet was washed with PBS and resuspended. Lymphocytes were stained with FITC-conjugated anti-CD4 and APC-conjugated anti-CD8 antibodies. T lymphocyte subset proportions (CD4^+^, CD8^+^) were quantified by flow cytometry (16).

### 2.11 Analysis of short-chain fatty acid metabolism

Fresh fecal samples (100 mg) from each rat group were accurately weighed into headspace vials (n = 6 per group). A 50 μL aliquot of 0.2% H_3_PO_4_ solution containing 4-methylvaleric acid internal standard (0.668 mg·mL^-1^) was added, and vials were immediately sealed. SCFAs analysis was performed using gas chromatography with headspace sampling (HS-GC). The headspace conditions were: sample heating at 80°C for 20 min, vial pressurization for 0.15 min, and injection for 0.5 min, with a total cycle time of 30 min and 10 min equilibrium time. The transfer line was maintained at 160°C.An Agilent DB-WAX capillaries column (30 m × 0.25 mm, 0.25 μm) was used to accomplish chromatographic separation. Splitless injection type was used, and the injected port temp was 250°C. The carrier gas was helium, which flowed at a steady 1.0 mL·min^-1^. The start point was 60°C; peak to 120°C at 30°C per minute; peak to 140°C at 5°C per minute (hold 1 minute); peak to 150°C at 10°C per minute (hold 1 minute); peak to 160°C at 5°C per minute (hold 1 minute); then peak to 230°Cat 35°C per minute and hold for 5 minutes. An optical mass spectrometer equipped with an electron ionization (EI) source running at 70 eV was used for the detection. The quadrupole was 150°C, the transfer line was 250°C, and the ion source was 230°C. A 4.5-minute solvent delay was used. Both full scan (SCAN) and selected ion monitoring (SIM) modes were used for data collecting across a mass range of 30–200 m/z.

### 2.12 16S rRNA gene sequencing of the gut microbiota

Experimental Procedure: Fecal samples from a randomly selected subset of mice (n = 6 per group) were subjected to microbial community profiling. Total genomic DNA was extracted using the Fecal Genome DNA Extraction Kit (AU46111-96, BioTeke, China) and quantified using a Qubit fluorometer (Invitrogen, USA). The V3-V4 hypervariable region of the bacterial 16S rRNA gene was amplified by PCR with universal primers 341F (5′-CCTACGGGNGGCWGCAG-3′) and 805R (5′-GACTACHVGGGTATCTAATCC-3′). The PCR protocol included an initial denaturation at 98 °C for 30 s, followed by 32 cycles of denaturation (98°C, 10 s), annealing (54°C, 30 s), and extension (72°C, 45 s), with a final extension at 72°C for 10 min. Amplicons were purified using AMPure XP Beads (Beckman Coulter, USA), re-quantified, and assessed for quality and size distribution using an Agilent 2100 Bioanalyzer (Agilent, USA). Libraries were quantified with Kapa Biosystems Illumina library quantitative kits, pooled in equimolar ratios, and sequenced on an Illumina NovaSeq 6000 platform to generate 2 × 250 bp paired-end reads (17).

Bioinformatic Analysis: Demultiplexed raw sequences were processed by removing sequencing primers with Cutadapt (v1.9) and merging paired-end reads using FLASH (v1.2.8). Quality control was performed with fqtrim (v0.94) to trim low-quality bases (Q< 20), discard short reads (< 100 bp), and remove reads containing > 5% ambiguous nucleotides. Chimeric sequences were filtered using Vsearch (v2.3.4). Amplicon Sequence Variants (ASVs) were inferred using DADA_2_ within QIIME_2_ (v2020.11). Taxonomy was assigned using the QIIME_2_ feature-classifier against the SILVA (v138) and NT-16S databases. Alpha-diversity (e.g., Observed ASVs, Shannon index) and beta-diversity (Bray-Curtis dissimilarity, visualized via PCoA) were calculated in QIIME2. Differential abundance at the genus level was assessed using the Wilcoxon rank-sum test (*P* < 0.05). Biomarkers were identified via LEfSe analysis (LDA score > 2.0, *P* < 0.05). All statistical analyses and visualizations were conducted using R software (v3.4.4).

### 2.13 Statistical analysis

Data are presented as mean ± standard deviation (SD). For *in vitro* studies, results are from three independent experiments (biological replicates). For *in vivo* studies, the sample size (n) for each analysis is specified in the corresponding figure legend and Methods section. Statistical analysis was performed using SPSS 27.0. Differences between two groups were assessed by Student’s t-test. Comparisons across multiple groups were analyzed by one-way analysis of variance (ANOVA) followed by Tukey’s *post hoc* test. A *P* value< 0.05 was considered statistically significant. Graphs were generated using GraphPad Prism (v9.0).

## 3 Results and discussion

### 3.1 Changes in polysaccharide content during the steaming process of *Cistanche deserticola*

The total polysaccharide content was determined by the phenol-sulfuric acid method. The calibration curve, constructed with glucose standards, was linear across the concentration range of 0 to 100 µg/mL. The regression equation was Y = 12.265X + 0.0203 (R² = 0.9994), where Y represents the absorbance at 490 nm and X is the glucose concentration (µg/mL). All sample measurements fell within this validated linear range. As illustrated in **Figure 1B**, the content of RCP was the lowest. With increasing processing time, the content of WCP-4, WCP-12, and WCP-16 gradually increased, with WCP-16 exhibiting the highest content. The increase in polysaccharide content was most pronounced from RCP to WCP-4, while the increase from WCP-12 to WCP-16 was more gradual, stabilizing at approximately 70%. The purity of the prepared polysaccharides was confirmed by quantitative analysis, showing low protein residue (< 3% for all samples, as determined by the Bradford assay) and high total sugar content (> 85% for all fractions). Detailed values for each sample are provided in **Supplementary Table S1**. The 1H NMR spectrum (**Supplementary Figure S1**) is indicative of α-glycosidic bonds, with a major signal observed in the anomeric proton region (δ 4.6–5.3 ppm).These results indicate that prolonged cooking times led to continuous high-temperature exposure, which altered the composition of the macromolecular polysaccharides. Complex chain-structured polysaccharides were decomposed into smaller polysaccharide and oligosaccharide components, thereby increasing the total polysaccharide content following processing.

This phenomenon of increased polysaccharide content after processing has also been observed in other traditional Chinese medicines such as *rehmannia* and *radix mesnili*. On one hand, the combination of damp heat and high temperature damages the cell wall structure of plants, promoting the release of polysaccharides (18), while also inactivating β-glucosidase and other enzymes, reducing the degradation of polysaccharides, resulting in a cumulative increase in total content (19). On the other hand, the 10%~20% ethanol content in the yellow wine acts as an organic solvent, promoting the hydroxylation of water-insoluble polysaccharides or glycosylated polysaccharides under high temperature, thereby enhancing water solubility (20). The changes in total polysaccharide content and efficacy of Cistanche during the wine-steaming process have not been reported in the literature. Modern research shows that the WCP can regulate the diversity of intestinal flora, increase the number of beneficial bacteria, enhance the production of short-chain fatty acids, promote the absorption of acteoside and improve the antioxidant capacity (21). Therefore, if the maximum pharmacological and therapeutic effects can be achieved, controlling the steaming time is particularly important.

### 3.2 Morphological observation

Scanning electron microscopy (SEM) was employed to analyze the morphology of RCP and WCP polysaccharides, revealing distinct patterns. As depicted in **Figure 1C**, at a magnification of ×500, RCP exhibited a dispersed block fragment structure with a few surface pores. At the same magnification, WCP-12 and WCP-16, obtained from different steaming durations, displayed pronounced surface fragments and pores. At a higher magnification of ×5000, RCP appeared as a cohesive unit with an uneven surface characterized by irregular protrusions and pores. The surface morphology of WCP changed significantly with increasing wine steaming time. Under prolonged or higher-temperature cooking conditions, the polysaccharide molecules began to fragment and aggregate, resulting in varying degrees of damage. The WCP-4, WCP-12, and WCP-16 polysaccharides were observed to have a massive structure with irregular fractures, a rough surface, and a loose texture. The longer the wine steaming time, the more pronounced the massive structure of the polysaccharides.

Microscopic infrared imaging (**Figure 1D**) intuitively demonstrated that the red regions of the polysaccharide powder gradually decreased, while the green regions increased, and the three-dimensional color map became more diverse. These microstructural changes are associated with the molecular weight of the polysaccharides, the type and quantity of monosaccharides, and the arrangement of monosaccharide molecules (10). Increased surface roughness is advantageous for enhancing biological activity and can improve intestinal flora and short-chain fatty acids (22).

### 3.3 Determination of molecular weight

The molecular weight distribution of polysaccharides significantly influences their physicochemical properties and biological activities. In this study, high-performance gel permeation chromatography coupled with a refractive index detector (HPGPC-RID) was employed to analyze the molecular weight distribution of *Cistanche deserticola* polysaccharides. The standard curve equation derived from HPGPC-RID was Y = -0.3917X + 9.6667 (R^2^ = 0.9995). The HPGPC-RID analysis results are depicted in **Figure 1E** and can be divided into five distinct regions. The first region corresponds to a retention time of 11.65 min and a molecular weight of 5,811,202.38 Da; the second region to 14.93 min and 378,531.48 Da; the third region to 19.03 min and 11,971.21 Da; and the fourth region to 21.19 min and 443.82 Da. The analysis reveals that the molecular weight of *Cistanche deserticola* polysaccharides decreased notably after wine processing. Specifically, the first region showed a gradual decrease, while the fourth region exhibited a gradual increase and a higher proportion. Moreover, the relative molecular weight distribution range of *Cistanche deserticola* polysaccharides narrowed with increasing steaming time.

Similar studies have shown that the molecular weight of polysaccharides change after processing. For example, the proportion of high molecular weight polysaccharides (> 1000 kDa) of *Polygonum multiflorum* Thunb gradually decreases after processing, while the proportion of low molecular weight polysaccharides (1–10 kDa) generally increases. Processing time affects the molecular weight distribution of polysaccharides, and the activity of polysaccharides will be affected by molecular weight, resulting in changes in the pharmacological activity of Polygonum multiflorum polysaccharides (23). The molecular weight of *Polygonum multiflorum*Thunb polysaccharide after processing is reduced, the structure is changed, and the anti-inflammatory and anti-cancer potential is enhanced (24). This trend may be related to the hydrolysis, degradation or structural reorganization of high molecular weight polysaccharides during wine steaming.

### 3.4 Monosaccharide composition

GC-MS indicated that the polysaccharides were primarily composed of glucose (Glu), galactose (Gal), mannose (Man), arabinose (Ara), rhamnose (Rha), and fructose (Fru), with a molar ratio of 48.89:6.76:1.01:1.35:2.61:3.24. Glucose was the most abundant monosaccharide (48.89%), followed by galactose (6.76%). The monosaccharide composition remained unchanged after wine processing. However, significant differences were observed in the molar ratios of the monosaccharides, indicating variations in their content. The molar ratios of the monosaccharides in the wine-processed polysaccharides (WCP) are detailed as follows:


WCP−4: Glu:Gal:Man:Ara:Rha:Fru=46.87:7.23:1.58:2.34:3.76:2.95

WCP−12: Glu:Gal:Man:Ara:Rha:Fru=44.35:8.58:2.43:2.01:5.74:2.39

WCP−16: Glu:Gal:Man:Ara:Rha:Fru=41.09:10.63:2.52:2.45:5.02:1.83

The proportions of glucose (48.89%→41.09%) and fructose (3.24%→1.83%) have significantly decreased, while the ratios of mannose (1.01%→2.52%) and galactose (6.76% →10.63%) have significantly increased. The proportions of arabinose (1.35%) and rhamnose (2.61%→5.02%) have remained relatively stable. This is consistent with the changes in the monosaccharide composition of *Cistanche deserticola* polysaccharides in previous studies (25). Mannose is widely used in health food development due to its significant efficacy in antiviral, anti-tumor, and prevention and treatment of urinary tract infections (26). High galactose content has various immunomodulatory activities and is applied in industries such as food, cosmetics, feed, and pharmaceuticals (27). Higher levels of mannose and galactose indicate stronger probiotic activity and better effects on immune regulation and the interaction between intestinal flora (28).

### 3.5 Effects of different steaming time on the immunomodulatory activity of *Cistanche deserticola* polysaccharide

#### 3.5.1 Effect of drug-containing serum of *Cistanche deserticola* polysaccharide on cell viability

**Figure 2:**
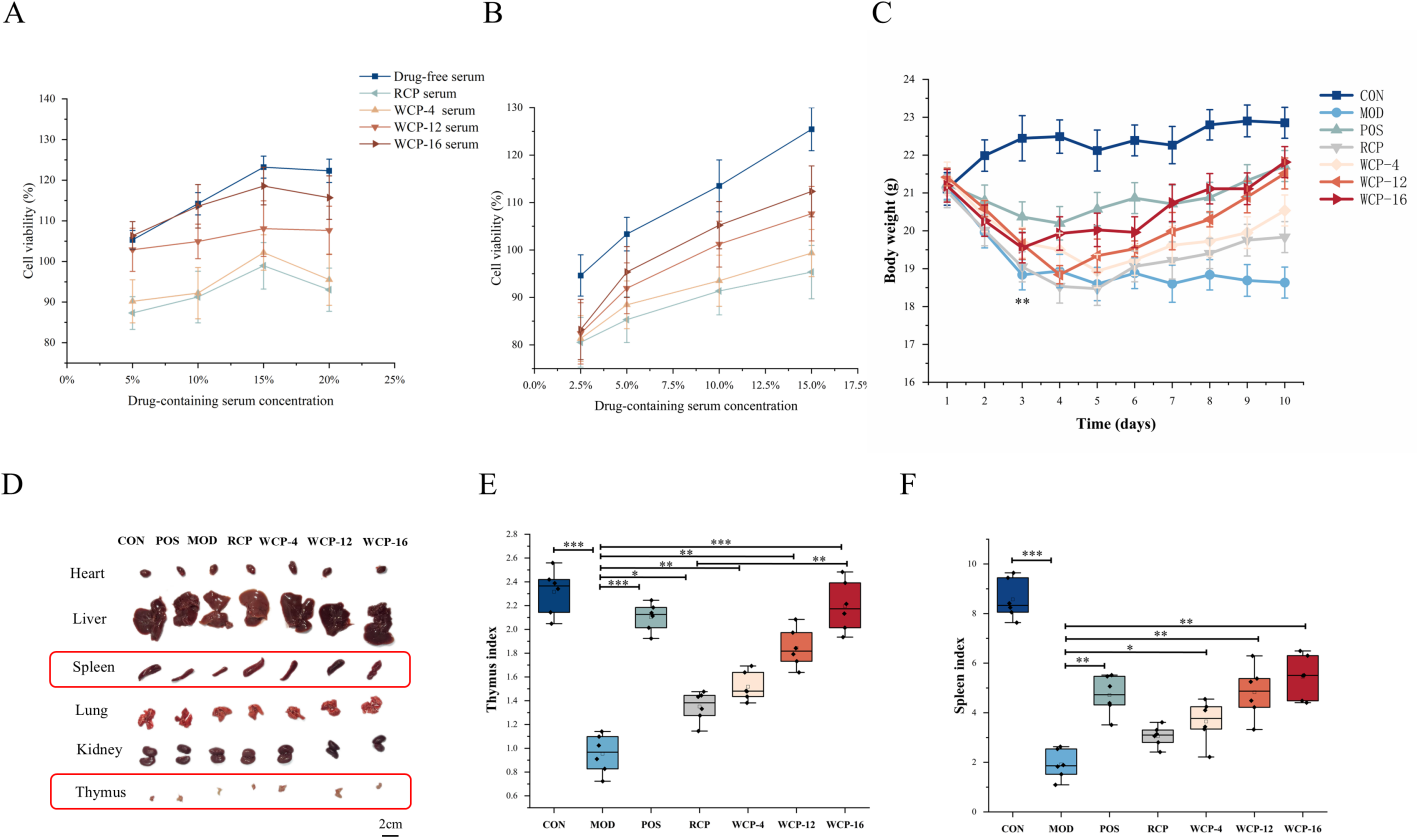
The effect of *Cistanche deserticola* polysaccharides on immune regulatory activity. **(A)** The influence of different concentrations of drug-containing serum on the viability of TM4 cells. **(B)** The influence of different concentrations of drug-containing serum on the viability of RAW264.7 cells. **(C)** Daily weight. **(D)** Schematic diagram of spleen and thymus. **(E)** Thymus index. **(F)** Spleen index. The data are presented as the mean ± standard deviation. *p < 0.05, **p < 0.01, ***p < 0.001.

The cytotoxic effects of different concentrations of drug-containing serum on RAW264.7 and TM4 cell viability were first evaluated using the CCK-8 assay. The results revealed that TM4 cell viability exceeded 80% across a serum concentration range of 5%-20%, whereas RAW264.7 cell viability remained above 80% within a concentration range of 2.5%-15% (**Figure 2A**). These findings indicate that RCP and WCP exert no cytotoxic effects on TM4 and RAW264.7 cells. In TM4 cells, serum containing WCP-16 at doses of 5%, 10%, 15%, and 20% significantly enhanced cell viability compared with RCP, WCP-4, and WCP-12 at equivalent doses. The drug-containing serum of WCP-16 markedly promoted TM4 cell viability. Similarly, in RAW264.7 cells (**Figure 2B**), the drug-containing serum of WCP-16 significantly enhanced cell viability.

#### 3.5.2 Effects of *Cistanche deserticola* polysaccharide on the body state, weight and immune organ index of immunosuppressed mice

During the acclimatization period, mice maintained on a standard diet exhibited healthy, shiny fur, brisk responses, and steady weight gain. Following cyclophosphamide administration, the mice experienced weight loss, dull and ruffled fur, lethargy, and sluggish movement, confirming the successful establishment of the immunosuppression model. As depicted in **Figure 2C**, aside from the control (CON) group, the body weight of mice in all other groups began to decline 24 hours after the start of modeling. After the third day of intragastric administration, the body weight of mice in the positive control (POS), RCP, WCP-4, and WCP-12 groups decreased gradually, while that of the WCP-16 group started to increase. The body weight trends of the WCP-16 and POS groups were similar, indicating that WCP-16 can mitigate the weight loss caused by cyclophosphamide-induced immunosuppression.

Immune organ indices, which are simple and commonly used indicators of immune status, reflect the immune level by measuring the relative weights of immune organs such as the thymus and spleen (29). The thymus and spleen play central roles in the development and maturation of T/B lymphocytes and in the execution of immune responses, respectively, and are thus referred to as “central immune organs” and “peripheral immune organs” (30). As shown in **Figure 2D**, immunosuppression led to tissue atrophy, with the thymus and spleen indices of the model (MOD) group being lower than those of any other group. The thymus and spleen indices of the WCP groups were higher than those of the RCP group (**Figures 2E, F**). The thymus index of the WCP-16 group was comparable to that of the CON group, indicating that WCP-16 has a significant capacity to restore immune organ function and reverse the decline in immune organ indices caused by cyclophosphamide, thereby preventing immune organ atrophy.

#### 3.5.3 Determination of cytokines and immunoglobulin content

**Figure 3:**
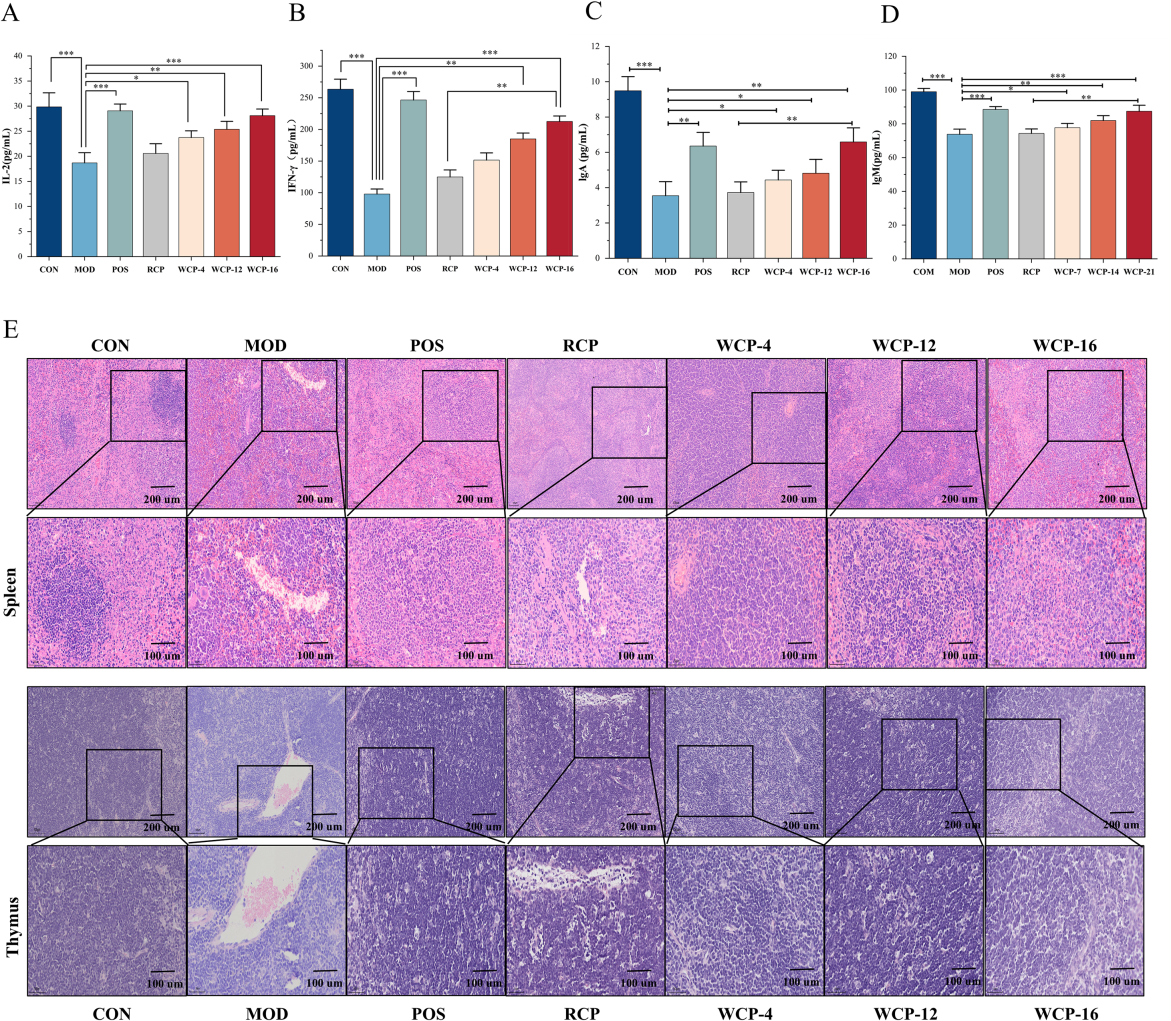
*Cistanche deserticola* polysaccharides’ effects on the levels of **(A)** IL-2, **(B)** IFN-γ, **(C)** IgA, and **(D)** IgM. The mean ± standard deviation is used to display the results. *p< 0.05, **p < 0.01, ***p < 0.001. **(E)***Cistanche deserticola* polysaccharide impact on the thymus and spleen tissue structure in mice (HE, ×200).

To assess the impact of RCP and WCP on the humoral immune system, we used ELISA to detect the amounts of immunoglobulins and inflammatory cytokines in the mouse blood. **Figures 3A-D** demonstrate that the MOD group’s levels of IL-2, IFN-γ, IgA, and IgM were considerably lower than the CON group’s (*p* < 0.05). After RCP and WCP treatments, their levels increased significantly with the extension of processing time: WCP-16 > WCP-12 > WCP-4 > RCP. This indicates that WCP-16 can significantly regulate cytokines and immunoglobulins to modulate immune activity. Immunoglobulins in particular are significant biomarkers of innate immunity, and immune-active cells produce cytokines. One important cytokine, IL-2, has several anti-tumor and immunomodulatory properties (20). The only type II interferon, IFN-γ, is released by NK cells and activated T cells, particularly Th1-type CD4^+^T cells and cytotoxic CD8^+^T cells. It has several important functions in both natural and innate immunity (31). Important elements of the immune system, IgM and IgA are essential for humoral and mucosal immunity. IgA is the immunoglobulin with the highest daily synthesis rate in adults and is the most important antibody in the mucosal immune system. IgM is the first type of antibody to appear during human individual development and immune response and plays an important role in the early stage of pathogen infection (32).

#### 3.5.4 HE staining of spleen and thymus

The spleen and thymus are not only important immune organs, but also two major hubs of the central and peripheral immune systems. The spleen is composed of red pulp, white pulp, and marginal zone. **Figure 3E** shows the histological morphology of the spleen. The spleen in the CON group exhibited an intact structure, with distinct boundaries between the white pulp and red pulp. In contrast, the spleen in the MOD group showed shrunken white pulp, excessive red pulp, loosely arranged cells, and the presence of vacuoles. After treatment with RCP and WCP-4, WCP-12, and WCP-16, the white pulp significantly increased, vacuoles decreased, and cells were more closely arranged, indicating an increase in lymphocytes. Notably, the spleen in the WCP-16 group was similar to that in the CON group. These results demonstrate that WCP-16 can effectively mitigate spleen damage caused by cyclophosphamide.

The thymus is composed of many lobules composed of cortex located at the edge of the lobule and medulla distributed in the center. In the CON group, thymocytes were neatly arranged, with a clear boundary between the cortex and medulla and minimal intercellular space. In contrast, the MOD group exhibited cortical atrophy, increased intercellular space in the medulla, and the presence of cell vacuoles. Following treatment with RCP, WCP-4, WCP-12, and WCP-16, the boundary between the cortex and medulla became distinct, cortical lymphocytes were abundant, and the morphological structure appeared normal, with no evident abnormalities in the medulla. The results in the WCP-16 group closely resembled those of the CON group, indicating that WCP-16 effectively repairs the thymus in immunosuppressed mice and alleviates cyclophosphamide-induced thymic damage.

#### 3.5.5 Effect on T lymphocyte subsets

**Figure 4:**
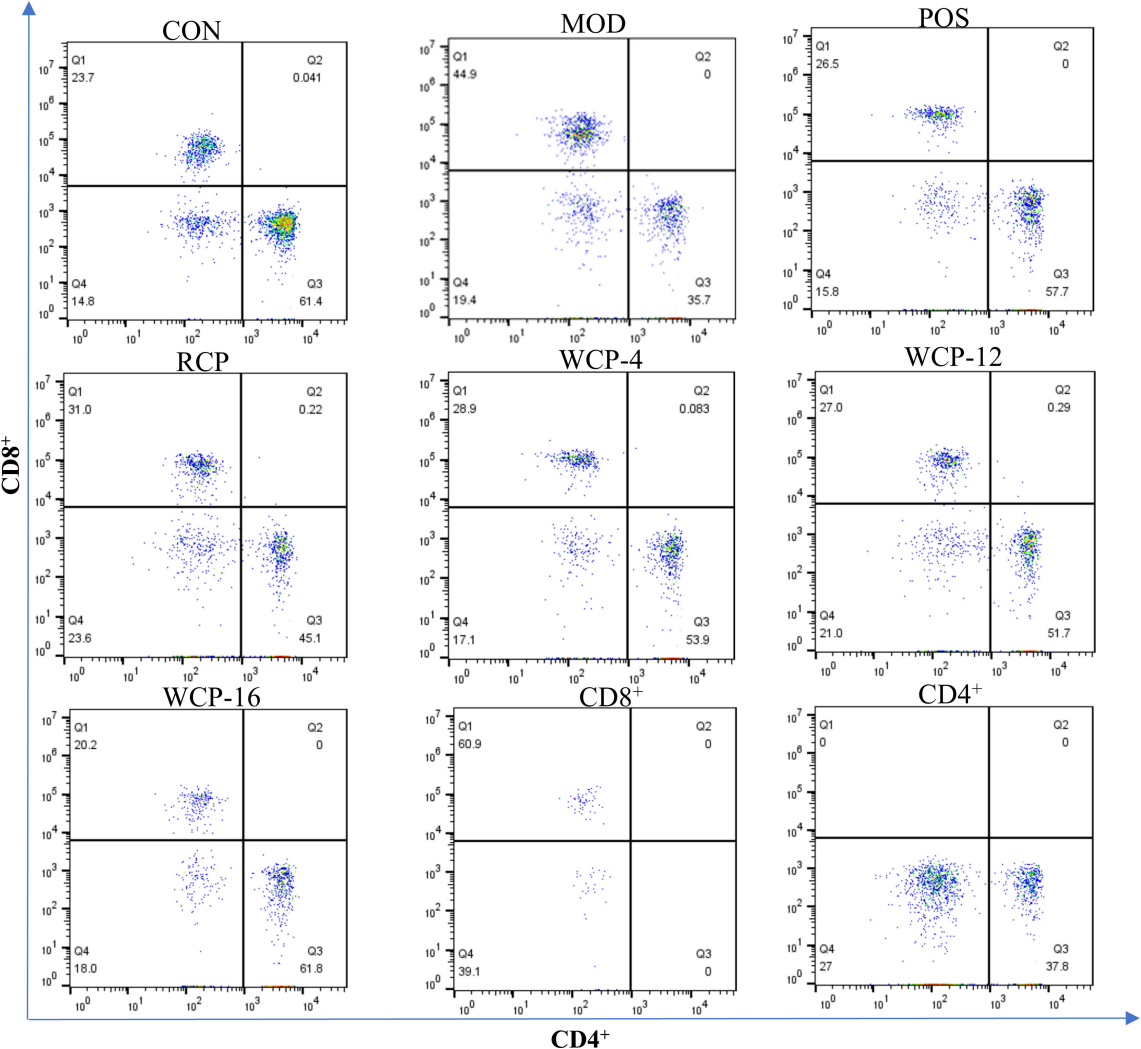
Effects of RCP, WCP-4, WCP-12 and WCP-16 on CD4^+^/CD8^+^ T lymphocytes in immunosuppressed mice.

To further explore the immunomodulatory effects of RCP and WCP on mouse lymphocytes, we analyzed their subgroups using flow cytometry. As shown in **Figure 4**, compared with the CON group, cyclophosphamide treatment led to a decrease in the number of CD4^+^ cells and a relative increase in CD8^+^ cells in the MOD group, resulting in a reversed CD4^+^/CD8^+^ ratio (i.e., less than 1.0). After treatment with RCP, WCP-4, WCP-12, and WCP-16, the CD4^+^/CD8^+^ ratio increased to 1.45, 1.86, 1.91, and 3.05, respectively. Notably, the recovery effect of the WCP-16 group was significantly higher than that of the POS group.

CD4^+^ and CD8^+^ T cells play crucial roles in the immune response. The proportions of CD4^+^ and CD8^+^ T cells and the CD4^+^/CD8^+^ ratio are important indicators of immune status (33). A significantly decreased CD4^+^/CD8^+^ ratio is associated with severe disease states, whereas an increased ratio indicates a positive regulatory state. The proportion of CD4^+^ T cells and the CD4^+^/CD8^+^ ratio were significantly reduced in the MOD group but were significantly increased after treatment with RCP and WCP, thereby alleviating cyclophosphamide-induced immunosuppression. These results demonstrate that RCP, WCP-4, WCP-12, and WCP-16 enhance immunity by regulating T lymphocyte differentiation. Moreover, the CD4^+^/CD8^+^ ratio of WCP gradually increased with processing time, indicating enhanced immune function and significant immunoregulatory effects.

### 3.6 The effects of RCP and WCP on short-chain fatty acids

In the intestinal tract, polysaccharides are digested and metabolized by the intestinal microbiota, generating short-chain fatty acids, such as acetic acid, propionic acid, and butyric acid, with health benefits. Short-chain fatty acids play a crucial regulatory role in the immune system, mainly manifested in regulating the activity and differentiation of immune cells, as well as maintaining the function of the intestinal barrier. SCFAs (especially butyric acid and propionic acid) inhibit the activity of histone deacetylases (HDAC), enhance histone acetylation at the Foxp3 gene locus, promote Treg cell differentiation, and inhibit excessive inflammation (34). Acetic acid promotes the secretion of IFN-γ by memory T cells, enhancing pathogen clearance; when the concentration reaches the threshold, it inhibits T-cell receptor (TCR) signaling, reduces excessive inflammation, and prevents tissue damage (35). Valeric acid activates the p38 MAPK and mTOR pathways, enhances the IL-10 secretion and anti-apoptotic ability of regulatory B cells (Breg), and inhibits T-cell inflammation in colitis models (36). The polysaccharides from *Cistanche deserticola* were not totally absorbed and metabolized during the digestive process in the present study. The intestinal microbes at the end of the colon primarily transformed them into short-chain fatty acids. In the presence of gastrointestinal microbes, such as SCFAs, different kinds of polysaccharides would produce different kinds of mediators. The intestinal flora and the generated SCFAs work together to control the organism’s wellbeing.

**Figure 5:**
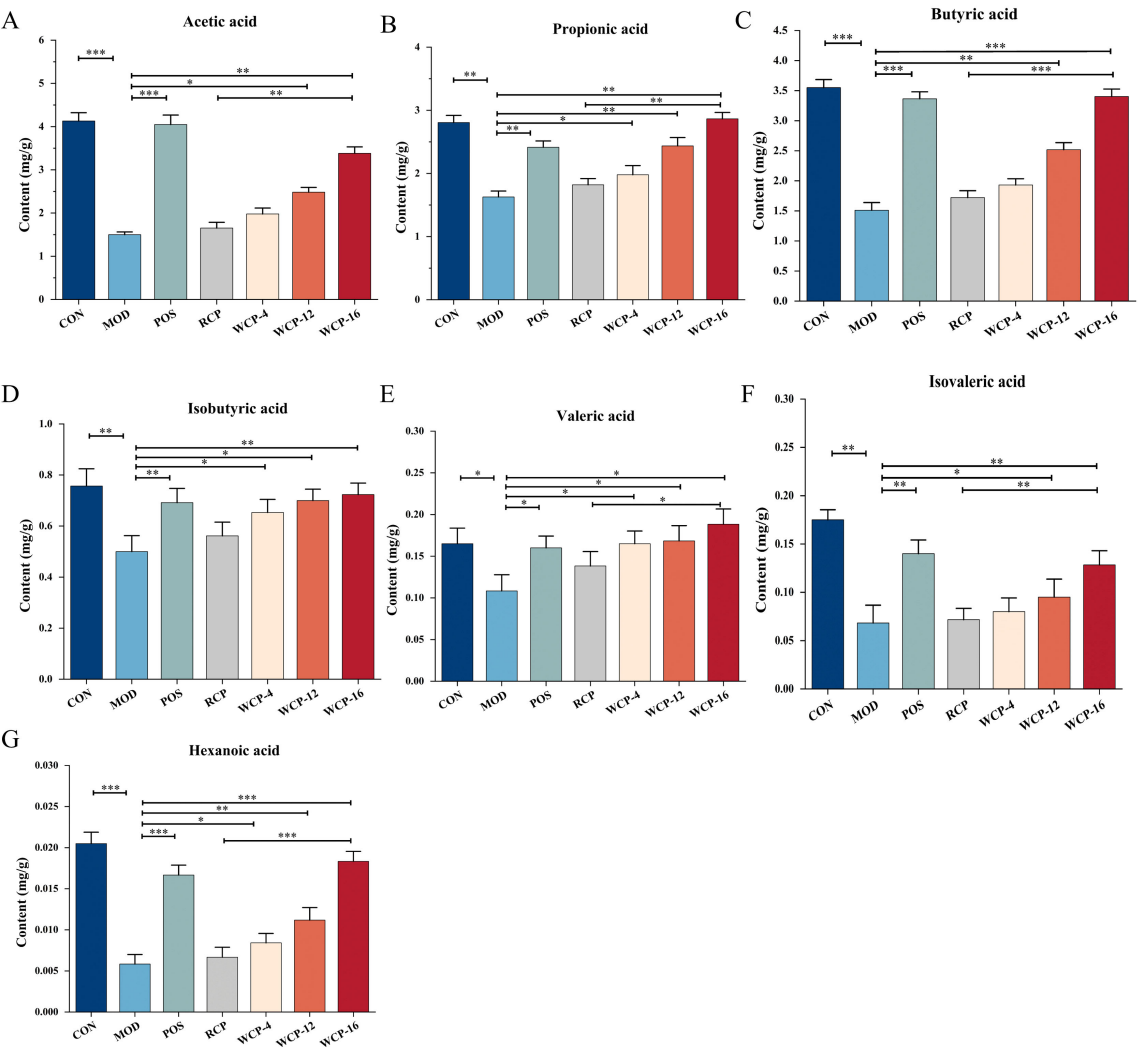
RCP and WCP’s effects on mouse stools’ SCFAs. **(A)**Acetic acid,**(B)** propionic acid,**(C)** butyric acid, **(D)**isobutyric acid, **(E)** valeric acid, and **(F)** isovaleric acid, **(G)** Hexanoic acid. Group differences indicated by different letters are statistically significant. The mean ± standard deviation is used to display the data (n = 8).*p < 0.05, **p < 0.01, ***p < 0.001.

**Figure 5** displayed the amount of SCFAs in the feces of each mouse group. Mice in the MOD group had considerably lower SCFA levels in their stool than mice in the CON group; the decreases were particularly pronounced for acetic acid, propionic acid, and butyric acid. It suggests that the amount of SCFAs in the intestinal tract of immunodeficient mice was considerably decreased by cyclophosphamide administration. Mice in each treatment group had significantly higher levels of acetic acids, propionic acid, butyric acid, isobutyric acid, valeric acid, and butyric acid among their feces (*p* < 0.05) than the MOD group. The mice in the WCP-12 and WCP-16 groups had more pronounced increases in SCFAs in their stool opposed to the RCP group, with the WCP-16 group’s rise being more pronounced than the WCP-12 group’s. Likewise, in mice with CTX-induced immunosuppression, polygona polysaccharose markedly raised the SCFAs concentration (37). The results have demonstrated that gut microbes may convert RCP and WCP into SCFAs, which would then have immunomodulatory benefits via the aforementioned pathways.

### 3.7 The effects of RCP and WCP on the gut microbiota of immunosuppressed mice

#### 3.7.1 Analysis of gut microbiota diversity

**Figure 6:**
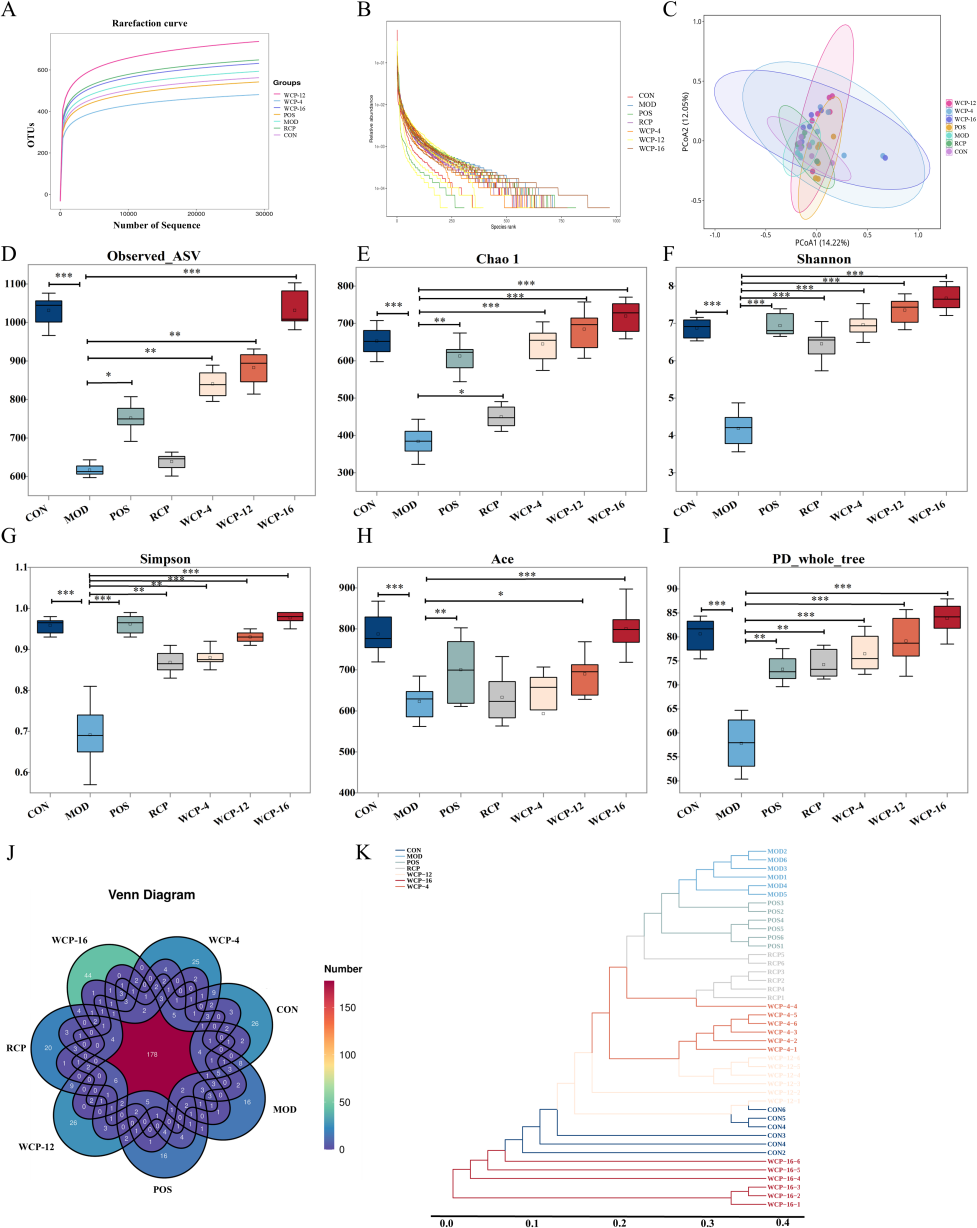
Analysis of the α and β diversity of the intestinal microbiota. **(A)** Sparse curve; **(B)** Rank abundance curve; **(C)** PCoA of the intestinal microbiota based on the weighted unifrac distance for β diversity; alpha diversity indices (including **(D)** observed microbial community abundance (ASV); **(E)** Chao1 index; **(F)** Shannon index; **(G)** Simpson index; **(H)** Ace index. **(I)** PD_whole_tree index; **(J)** Venn diagram; **(K)** UPGMA clustering analysis. Data are presented as mean ± standard deviation (SD), n = 6. *p < 0.05, **p < 0.01, ***p < 0.001.

Gut microbiota diversity is an essential ecological parameter for maintaining host immune homeostasis. The decrease of diversity will lead to the synergistic imbalance between innate and adaptive immunity and increase the risk of chronic inflammation and autoimmune. Its abundance directly determines the dynamic balance between immune tolerance and response (38). The intestinal microbial diversity of mice was analyzed using the Illumina NovaSeq 6000 sequencing platform. The dilution curve indicated whether the sample data volume was sufficient. **Figure 6A** shows an upward trend that stabilizes with increasing sequencing quantity, indicating sufficient sequencing data for analysis without enhancing the probability of new species emergence. **Figure 6B** displays a smooth curve, reflecting high species abundance and even distribution in the sample, confirming the adequacy of the sequencing data for subsequent analysis. Alpha diversity analysis was performed to compare the abundance and diversity of intestinal flora among different groups of mice. As shown in **Figures 6D–I**, the MOD group exhibited significantly reduced bacterial abundance (ASV), Chao1, Shannon, Simpson, Ace, and PD_whole_tree indices (*p* < 0.001). Compared with the MOD group, these indices were improved to varying degrees in the treatment groups, with WCP-16 and RCP showing significant effects, indicating their potential to enhance the diversity and abundance of intestinal flora in mice.

β diversity analysis using PCoA and hierarchical clustering revealed distinct clustering patterns among groups. As shown in **Figure 6C**, the CON, POS, and treatment groups exhibited clear separation from the MOD group, indicating significant differences in microbial community structure. This suggests that cyclophosphamide alters the composition of the intestinal microbiota. After RCP and WCP intervention, the microbiota structure shifted closer to that of the CON group, with the WCP-16 group showing the most significant separation from the MOD group, indicating superior efficacy in restoring the microbiota disrupted by cyclophosphamide. The Venn diagram was used to compare the common and unique microbial communities between different groups and quantify the degree of microbial community sharing. UPGMA clustering analysis revealed that the intestinal microbiota in the WCP-16 group was closer to the normal state. The RCP and POS groups clustered together, followed by clustering with the WCP-4 and WCP-12 groups, indicating that both RCP and WCP have regulatory effects on the intestinal microbiota (**Figures 6G, K**).

#### 3.7.2 The effects of RCP and WCP on the gut microbiota

**Figure 7:**
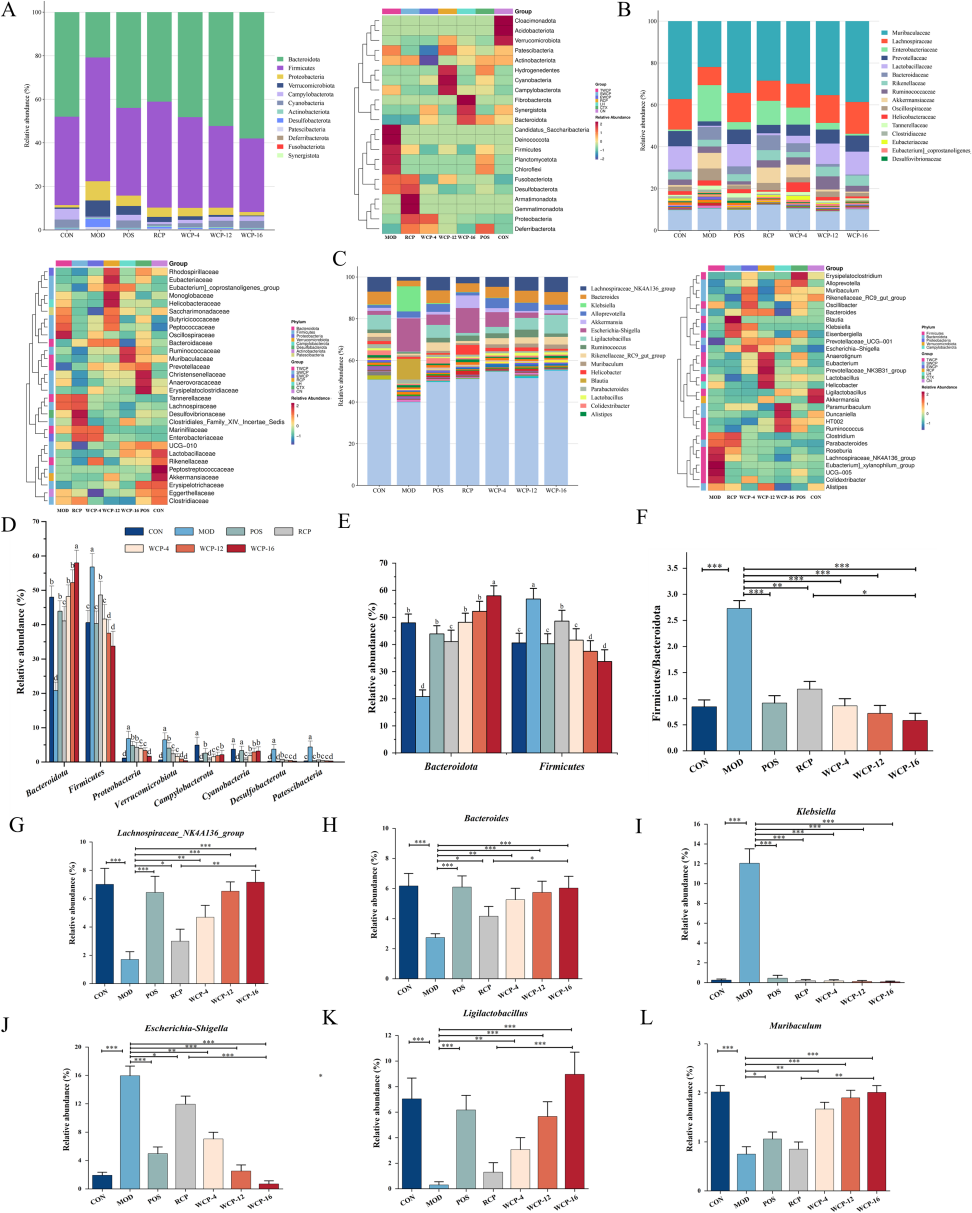
Illustrates how the gut microbial ecology is affected by immunosuppressed mice (n = 6). **(A)** A phylum classification level bar chart and heatmap assessment of the bacterial community in the mouse intestine. **(B)** Level of classification bar chart and heatmap assessment of the microbial population in the mouse intestines. **(C)** Heatmap and bar chart assessment of the mouse intestinal microbial population at the genus classified degree. **(D)** A phylum-level comparison of the relative abundance of eight intestinal bacteria in the gut of each mouse group. **(E)** A phylum-level comparison of the relative abundance of the Bacteroidetes and Firmicutes phylums in the intestines of each mouse group. **(F)** A comparison between each group’s Firmicutes to Bacteroidetes (F/B) ratio. **(G-L)** A comparison of the relative abundance of gut bacteria at the genus level among each mouse group. Mean ± standard deviation (SD), n = 6,*p < 0.05, **p < 0.01, ***p< 0.001.

As the largest and most complex immune organ of the human body, the intestinal tract is the most densely populated area of the body. These intestinal microorganisms play a key role in nutrient absorption, metabolic regulation and immune function (39). Intestinal microbiota produce bioactive SCFAs by fermenting indigestible polysaccharides from dietary sources. This metabolic process constitutes the core link of the polysaccharide-bacteria- metabolite-host regulatory axis, which has multiple physiological significance for maintaining body health. In addition to the regulation of intestinal microbial diversity, RCP and WCP can also regulate the structure of intestinal flora in immunosuppressed mice (**Figures 7A-C**).The phylum-level variations in microbiota abundance, as illustrated in **Figure 7D**, suggest that cyclophosphamide leads immune-deficient mice to have higher relative abundances of *Firmicutes*, *Proteobacteria*, *Verrucomicrobiota*, *Desulfobacterota*, and *Patescibacteria* and lower relative abundances of *Bacteroidota*, *Campylobacterota*, and *Cyanobacteria*. The percentage of the relative abundance of the *Firmicutes* phylum compared to that of the *Bacteroidetes* phylum is known as the F/B ratio (*Firmicutes/Bacteroidetes* ratio), and it is used as a standard for assessing the balance of the gut microbiota. It is considered one of the macroscopic markers for assessing gut microecology balance and risk of illness (40).

Immune system disorders and metabolism-related processes are linked to changes in this ratio. (**Figure 7E**) A discrepancy in the F/B ratio can result in a number of illnesses, including bowel inflammation and atherosclerotic linked to obesity (41). Intestinal flora imbalance resulted from the MOD group’s higher *Firmicutes* phylum abundance and lower *Bacteroidetes* phylum abundance, as well as a markedly elevated F/B ratio. Following RCP and WCP therapy, the percentage of the *Bacteroidetes* phylum greatly rose, whereas the percentage of the *Firmicutes* phylum significantly declined. Furthermore, compared to the MOD, the F/B ratios in the POS group, RCP group, and WCP-4, WCP-12, and WCP-16 treatment groups were substantially lower. *Campylobacterota* and *Cyanobacteria* (27) can create a local anaerobic environment to activate the [NiFe]-hydrogenase of cyanobacteria, promoting the production of hydrogen (H_2_), continuously clearing elevated reactive oxygen species and reducing inflammatory factors, thereby reducing the production of inflammation. However, the content in the MOD group decreased. More and more data have identified the *Proteobacteria* phylum as a possible microbial characteristic of diseases.

According to studies, metabolic problems and inflammatory bowel illness are linked to a surge in the amount of members of this phylum. Serum levels of the cytokines associated with inflammation TNF-α, IL-1β, and IL-2 have a positive correlation with the abundance of the degrading bacterial group *Desulfobacterota*. Cognitive function deteriorates and the inflammatory response is exacerbated when the *Desulfobacterota* group’s density rises (42). The polysaccharides restored the variety of the intestinal microbiota and improved the functioning of the intestinal barrier in the colitis animal model caused by dextran sulfate sodium (DSS) by decreasing the abundance of *Patescibacteria* and *Verrucomicrobiota*. protecting against chronic gastrointestinal inflammation by controlling the immunological response, intestinal microbiota, and metabolic homeostasis (43). At the family level (**Figure 7B**), the abundance of *Muribaculaceae*, *Lachnospiraceae*, *Prevotellaceae*, *Lactobacillaceae* in the MOD group was lower, and the abundance of *Enterobacteriaceae* and *Bacteroidaceae* was higher. Each administration group will show different degrees of negative correlation with the MOD composition. The *Bacteroidetes* includes the *Muribaculaceae* family of bacteria. In addition to inhibiting low-grade inflammation through the intestinal-liver system, this probiotic can produce SCFAs like acetic acid, propionic acid, and butyric acid. It additionally generates vitamins for the host, such as vitamin B_1_ (thiamine), vitamin B_2_ (riboflavin), vitamin B_3_ (nicotinic acid), vitamin B_5_ (pantothenic acid), and vitamin B_7_ (biotin) (44). *Lachnospiraceae* and *Prevotellaceae* (45), as specific intestinal bacteria, produce butyrate short-chain fatty acids, which can restore immune homeostasis by enhancing the immune regulation mediated by regulatory T cells (Treg). *Lactobacillaceae*, as the most widely used and widely studied probiotics, can improve the tight junction of intestinal epithelial cells, maintain epithelial integrity and reduce inflammation (46). *Enterobacteriaceae* is a multi-drug resistant harmful bacterium, in which Escherichia coli can cause enteritis and systemic infection by invading intestinal mucosal cells (47). *Bacteroidaceae* pathogen-mediated ferroptosis induces systemic inflammation and hematopoietic toxicity (48).

The representative diagrams of the changed bacterial genera are shown in **Figures 7G-L**. The contents of *Lachnospiraceae_NK4A136_group*, *Bacteroides*, *Muribaculum*, and *Ligilactobacillus* decreased in the MOD group, while the contents of *Klebsiella* and *Escherichia-Shigella* increased. On the contrary, in the groups treated with various polysaccharides, the contents of *Lachnospiraceae_NK4A136_group*, *Bacteroides*, *Muribaculum*, and *Ligilactobacillus* increased, while the contents of *Klebsiella* and *Escherichia-Shigella* decreased. *Lachnospiraceae_NK4A136_group*, *Bacteroides*, and *Muribaculum* play an important role in promoting the production of SCFAs, enhancing peripheral immunity, regulating the intestinal immune microenvironment, and treating immunosuppressive diseases (49). More and more evidence emphasizes that *Ligilactobacillus* is involved in host intestinal metabolism and immune activity as a probiotic, and its abundance is closely related to intestinal health (50). *Klebsiella*, as an intestinal pathogen, the polysaccharide in dietary fiber increases the colonization of *Klebsiella* pneumoniae, reduces the diffusion of inflammatory mediators, and reduces sepsis (51). *Escherichia-Shigella* is characterized by intestinal dysbiosis. *Escherichia-Shigella*’s notable growth is indicative of IgA nephropathy in IgAN individuals and could be a helpful biomarker for IgAN evaluation and therapy. In contrast to the RCP group, the WCP group was able to decrease the abundance of *Klebsiella* and *Escherichia-Shigella* while considerably increasing the abundance of *Lachnospiraceae_NK4A136_group*, *Bacteroides*, *Muribaculum*, and *Ligilactobacillus*. Thus, by raising the proportion of predominant bacterium in intestinal flora, WCP-16 decreased the proliferation of harmful bacteria. Spearman correlation analysis revealed significant associations between WCP-modulated bacterial genera, short-chain fatty acid (SCFA) levels, and systemic immune markers (**Supplementary Figure 2**), providing supportive data for the functional links within the gut microbiota–metabolite–immune axis. This study demonstrates that rice-wine processing significantly enhances the immunomodulatory activity of *Cistanche deserticola* polysaccharides through structural optimization. The modified polysaccharide structure may exert dual mechanisms: first, by enhancing interactions with pattern recognition receptors on immune cells, thereby activating downstream signaling pathways; second, by improving fermentability by specific probiotics, promoting the production of SCFAs (particularly butyrate), which in turn strengthen the intestinal barrier and modulate systemic immunity via receptors These findings provide a scientific basis for traditional processing methods and suggest that structurally optimized polysaccharides may serve as potential functional modulators of the gut–immune axis. Future research should explore several directions: First, fecal microbiota transplantation experiments are needed to establish causality between WCP-modulated microbiota and immune outcomes. Second, the specific pathways mediated by metabolites should be clarified using techniques such as SCFA receptor antagonists. Furthermore, the broad efficacy of WCPs should be validated in various immune dysregulation models (e.g., autoimmunity, aging-related immunosenescence). Ultimately, systematically elucidating the processing–structure–microbiota–immune axis will provide a comprehensive theoretical framework and practical pathway for developing precision immunomodulators derived from traditional medicinal materials.

## 4 Conclusion

Based on the findings of this study, the polysaccharide content of *Cistanche deserticola* increased with prolonged steaming time, while the molecular weight decreased and the monosaccharide composition profile changed significantly. Concurrently, alterations in its external morphological structure were observed. Investigations into its biological activity and immunomodulatory properties revealed that polysaccharides obtained after longer steaming durations exhibited enhanced protective effects in cyclophosphamide-induced immunosuppressed mice, concomitant with modulation of the gut microbiota. Specifically, both RCP and WCP administration were associated with the recovery of body weight and immune organ indices, elevation in serum levels of IFN−γ, IL−2, IgA, and IgM, increased fecal concentrations of SCFAs, and a partial restoration of gut microbial community structure toward that of healthy controls. Collectively, these data indicate that both RCP and WCP can significantly improve systemic immune parameters in this model. The effects appeared most pronounced after 16 hours of steaming, as reflected by the superior immunomodulatory efficacy of the WCP−16 fraction. It should be noted that the present evidence primarily demonstrates correlative relationships; future studies employing fecal microbiota transplantation (FMT) and SCFAs receptor antagonism are warranted to establish direct causal mechanisms linking the observed microbial and metabolic changes to the immunomodulatory outcomes.

## Data Availability

The original contributions presented in the study are included in the article/**Supplementary Material**. Further inquiries can be directed to the corresponding authors.
