# Supplementary material for: Impact of rice wine-steamed Cistanche deserticola polysaccharides on intestinal flora and immunological modulation in immunosuppressive mice induced by cyclophosphamide
Source: Front Immunol. 2026 Feb 5;17:1732818. doi: 10.3389/fimmu.2026.1732818 (PMC12916368; doi:10.3389/fimmu.2026.1732818)
Supplement: Supplementary file 1 [file DataSheet1.docx]

Supplementary Material

# Supplementary Tables

**Supplementary Table S1.** Characterization of Polysaccharide Samples

| Sample | Total Sugar Content (%)¹ | Protein Content (%)² |
| --- | --- | --- |
| RCP | 86.03 | 2.57 |
| WCP-4 | 86.35 | 2.66 |
| WCP-12 | 87.45 | 2.38 |
| WCP-16 | 87.61 | 2.72 |

Footnotes:

¹ Total sugar content was determined by the phenol‑sulfuric acid method using glucose as a standard.² Protein content was determined by the Bradford assay using bovine serum albumin (BSA) as a standard.

# Supplementary Figures


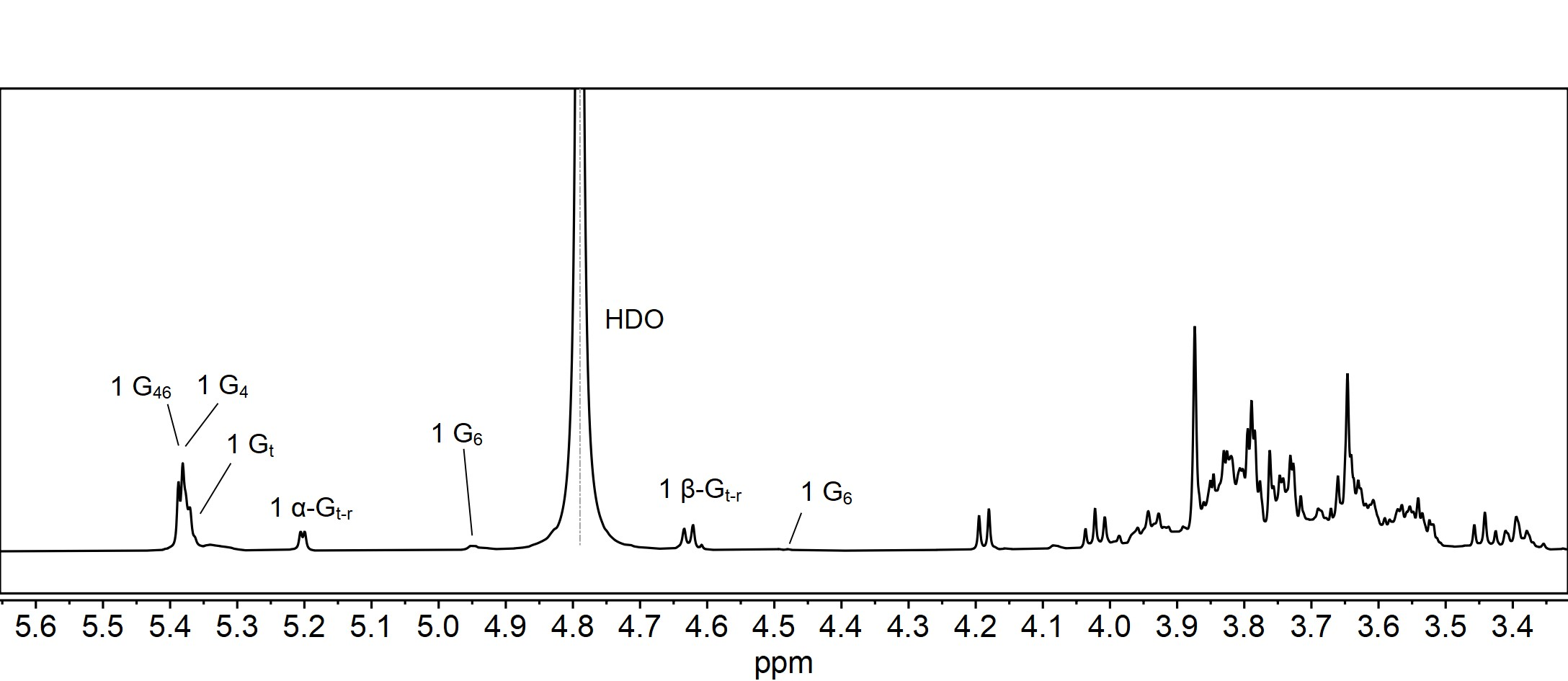


**Supplementary Figure 1.** *Cistanche deserticola* Polysaccharide 1H NMR spectrum

A

B

C


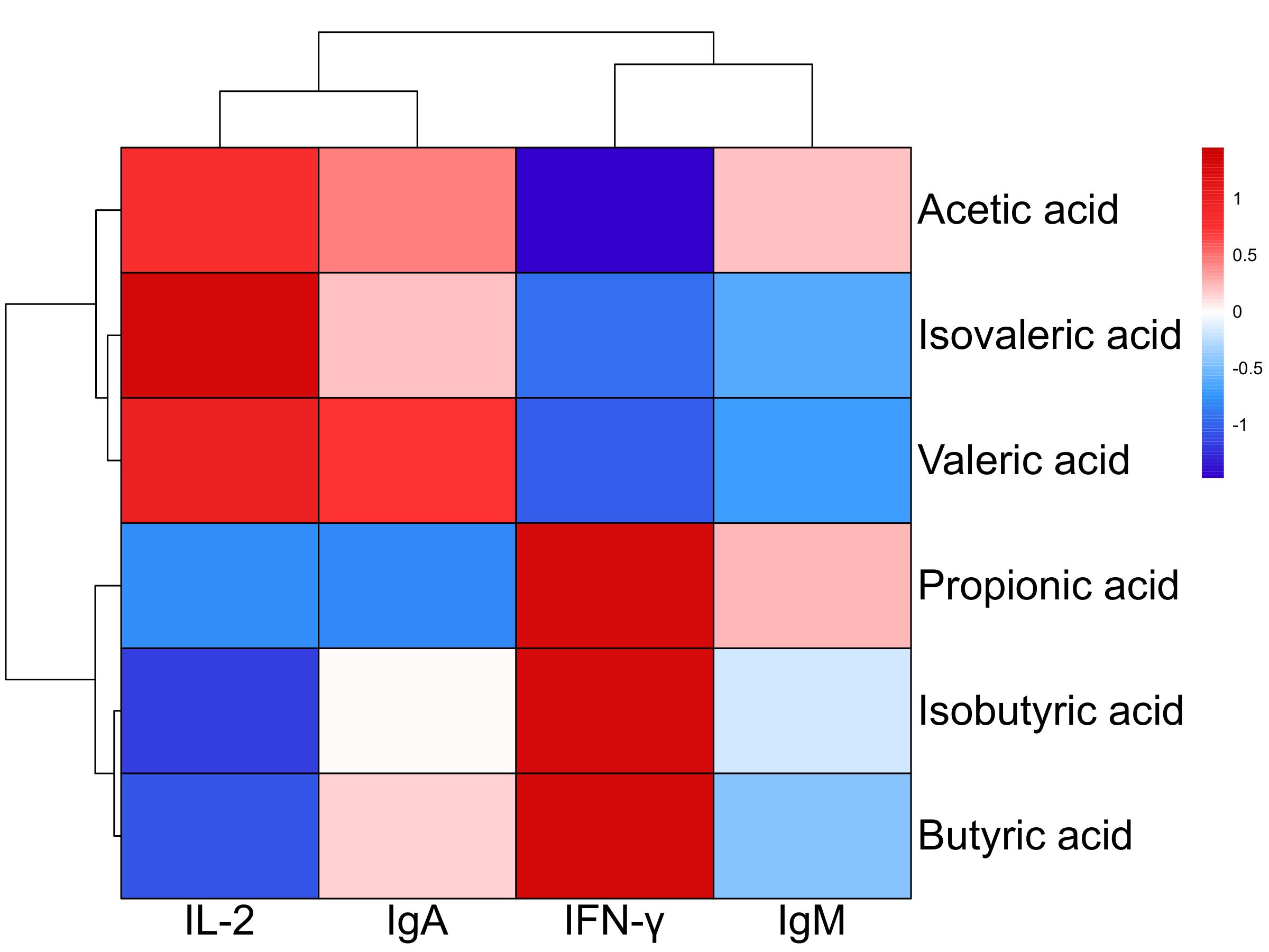

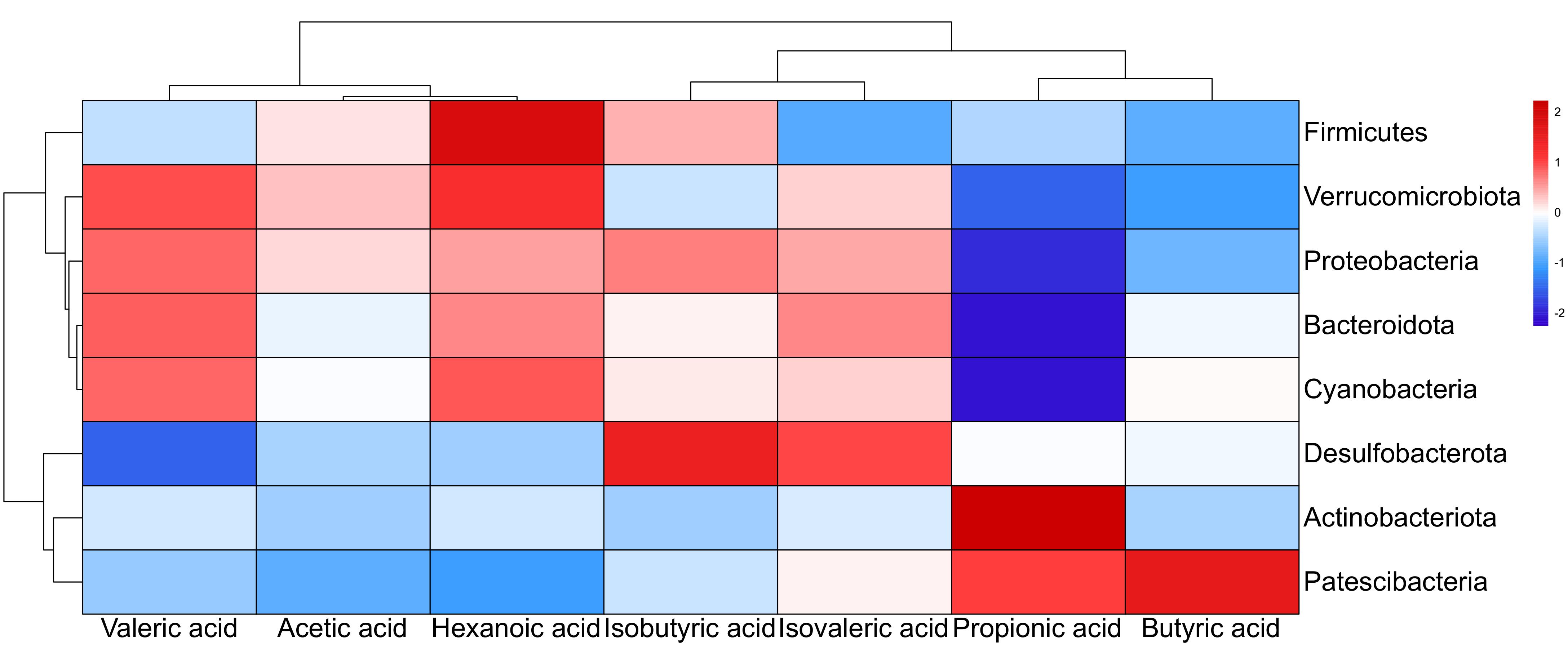

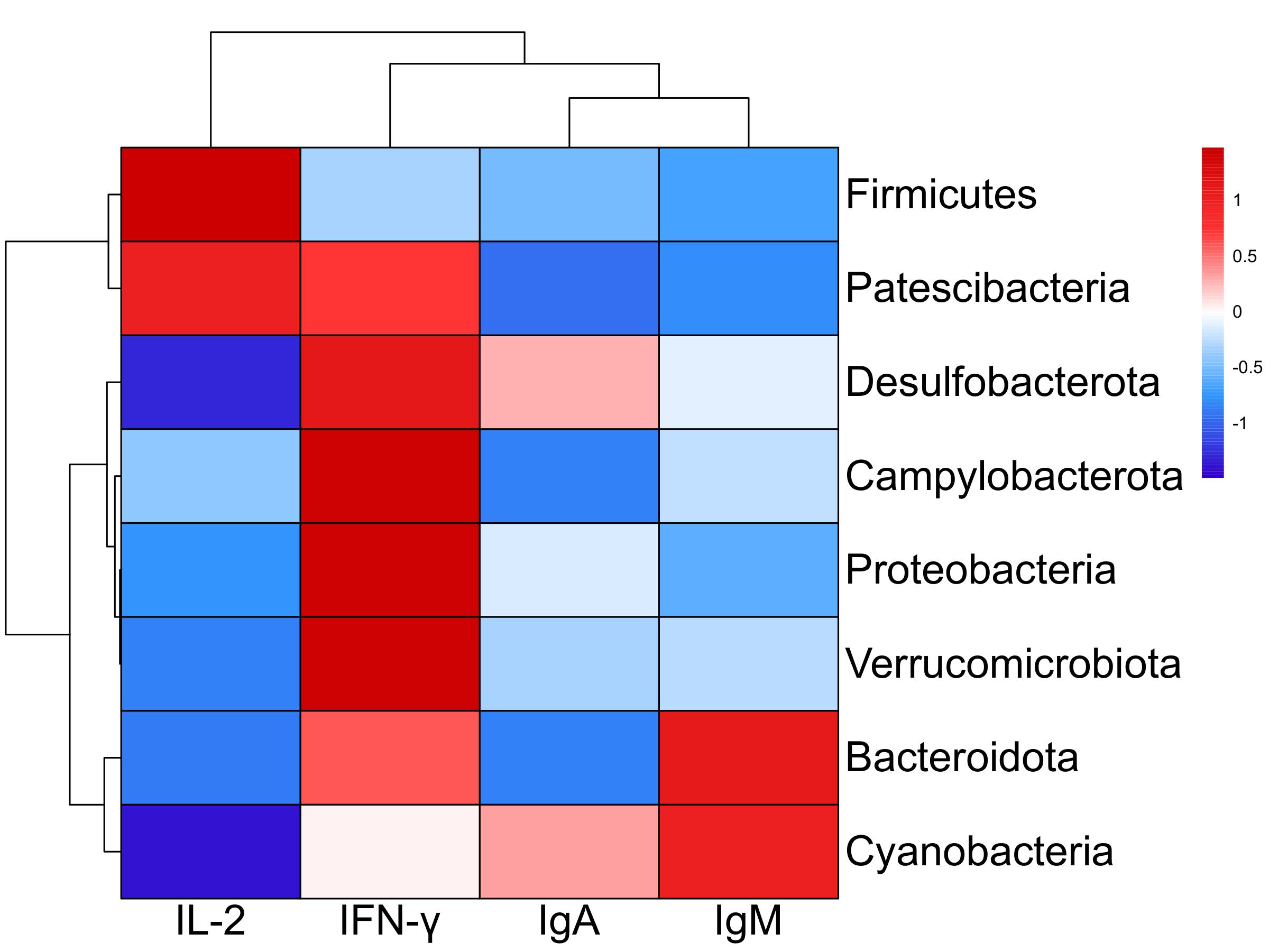


**Supplementary Figure 2.** Correlation analysis heatmap. (A) Correlations among short-chain fatty acids (SCFAs) and immune/biochemical parameters. (B) Correlations between immune/biochemical parameters and gut microbiota (at the genus level). (C) Correlations between SCFAs and gut microbiota (at the genus level).
